# A biomathematical model of SARS-CoV-2 in Syrian hamsters

**DOI:** 10.1038/s41598-024-80498-9

**Published:** 2024-12-18

**Authors:** Sibylle Schirm, Geraldine Nouailles, Holger Kirsten, Jakob Trimpert, Emanuel Wyler, Luiz Gustavo Teixeira Alves, Markus Landthaler, Peter Ahnert, Norbert Suttorp, Martin Witzenrath, Markus Scholz

**Affiliations:** 1 Institute for Medical Informatics, Statistics and Epidemiology (IMISE), University of Leipzig, 04107 Leipzig, Germany; 2 Department of Infectious Diseases and Respiratory Medicine and Critical Care, Charité – Universitätsmedizin Berlin, Corporate Member of Freie Universität Berlin and Humboldt-Universität zu Berlin, 10117 Berlin, Germany; 3 Institute of Virology, Freie Universität Berlin, 14163 Berlin, Germany; 4 Berlin Institute for Medical Systems Biology (BIMSB), Max Delbrück Center for Molecular Medicine in the Helmholtz Association (MDC), 10115 Berlin, Germany; 5 Institute for Biology, Humboldt-Universität zu Berlin, 10099 Berlin, Germany; 6 German Center for Lung Research (DZL), Berlin, Germany

**Keywords:** SARS-CoV-2, Mathematical model, Immune response, T-cells, Macrophages, Natural killer cells, B cells, Neutralising antibodies, CCL8, CXCL10, Viral infection, Viral infection, Computational models, Differential equations, Systems biology

## Abstract

When infected with SARS-CoV-2, Syrian hamsters (Mesocricetus auratus) develop moderate disease severity presenting key features of human COVID-19. We here develop a biomathematical model of the disease course by translating known biological mechanisms of virus-host interactions and immune responses into ordinary differential equations. We explicitly describe the dynamics of virus population, affected alveolar epithelial cells, and involved relevant immune cells comprising for example CD4+ T cells, CD8+ T cells, macrophages, natural killer cells and B cells. We also describe the humoral response dynamics of neutralising antibodies and major regulatory cytokines including CCL8 and CXCL10. The model is developed and parametrized based on experimental data collected at days 2, 3, 5, and 14 post infection. Pulmonary cell composition and their transcriptional profiles were obtained by lung single-cell RNA (scRNA) sequencing analysis. Parametrization of the model resulted in a good agreement of model and data. The model can be used to predict, for example, the time course of the virus population, immune cell dynamics, antibody production and regeneration of alveolar cells for different therapy scenarios or after multiple-infection events. We aim to translate this model to the human situation in the future.

## Introduction

Due to continued high disease burden, there is pressing need to understand the pathomechanisms of SARS-CoV-2. This includes mechanistic disease models to simulate and predict new therapy paradigms or schedules. Difficulties lie in the high complexity of immune responses following SARS-CoV-2 infection and the heterogeneity of disease manifestations in patients, reaching from no or mild symptoms to life-threatening disease conditions.

The quality and accuracy of prospective biomathematical models of virus-host interactions depend on the quality and level of detail provided by the input data. A challenge remains here that particularly human lung tissue, representing the site of infection, can usually only be sampled post mortem. Often, only viral load data are available from patients to inform model parameters. Despite these limitations, some in-host models have been developed since the disease emerged in 2019. For example, Hernandez-Vargas and Velasco-Hernandez^1^ reviewed existing work comprising models of target cells only or simple models of immune response. Model results were partly compared with patient’s viral load data from literature resources. Another target cell limited model can be found in Abuin et al.^2^, and the effect of different antivirals on viral load is modelled in^3^. Several modelling approaches were also discussed in Perelson and Ke^4^. The modelling of Sanche et al.^5^ considers infected cells carrying pathogen-associated molecular patterns and damaged cells producing damage-associated molecular patterns regarding severe or mild disease progression. Models of SARS-CoV-2, MERS-CoV, and SARS-CoV with different therapy approaches are analysed and compared with patient’s viral load data in Kim et al.^6^.

The model analysed by Almocera et al.^7^ describes interactions of the virus and effector T cells. Du and Yuan^8^ proposed a model of COVID-19 including interactions between host innate and adaptive immune responses without experimental data and compared it with an influenza virus disease model. The impact of different virus-host interactions on the outcome was also modelled in Sahoo et al.^9^ without supporting data.

Mochan et al. study an ordinary differential equation model of interactions between virus, infected and healthy epithelial cells in the upper and lower respiratory tracts, global levels of pro-inflammatory and anti-inflammatory mediators, and damage. The effects of anti-inflammatory and antiviral drugs were simulated. Parameters were fitted to experimental data (viral RNA levels in nasal cavity and lungs, IL-15, and clinical symptoms scores from rhesus macaques.

Reis et al.^10^ propose a comprehensive model of immune response and cytokine release in severe disease, supported by patient data of viral load, IL-6, IgG and IgM. Voutouri et al.^11^ developed a comprehensive model of SARS-CoV-2 infection, including the renin-angiotensin system and ACE2, viral load, immune cells, cytokines, oxygen saturation and the coagulation cascade. This model is compared with clinical data of serum Ang-2 and IL6 levels, neutrophils, $\text {CD8}^+$ T cells, and viral load.

Li et al.^12^ developed a within-host viral dynamic model of SARS-CoV-2, compared results with chest radiograph score data and estimated the basic reproductive number in hosts. This model is further analysed by Nath et al.^13^. Sumi and Harada^14^ presented a comprehensive model of SARS-CoV-2 infection including innate and adaptive immunity. This model considers age-related risk factors for the development of severe COVID-19, supported by patients viral load and immunoglobulin concentration data from the literature. Another model of innate and adaptive immune response, antiviral treatment and vaccination is proposed by Ghosh^15^. Model results were again compared to viral load data from the literature.

Challenger et al.^16^ described a mechanistic model of the upper respiratory viral load dynamics during SARS-CoV-2 based on viral load data from the literature. A model of interactions between susceptible epithelial cells, infected epithelial cells, viral load, natural killer cells, and T-lymphocytes is analysed by Chowdhury^17^. Chatterjee et al.^18^ modelled infected cells, the strength of CD8 T-cell response, the strength of the cytokine-mediated innate immune response, and tissue damage in mild or severe disease course. The model was adapted to time series of viral load data from human and can explain heterogeneity of infection outcomes. Sazonov et al.^19^ develop a stochastic model based on a Markov Chain Monte Carlo approach to analyze statistical characteristics of the SARS-CoV-2 life cycle, including the probability for a non-degenerate infection process and the effects of IFN and ACE2 binding affinity. Grebennikov et al.^20^ developed an ordinary differential equation model of the SARS-CoV-2 intracellular life cycle based on cell culture data, which provides a kinetic description of the major replication stages of SARS-CoV-2 and the identification of the life cycle stages that have the strongest impact on viral replication. An agent based model, supported by patients viral load data, is presented by Moses et al.^21^. Getz et al.^22^ present an open-source community approach to SARS-CoV-2 modelling including several immune cells and cytokines. Sego et al.^23^ build an open-source, extendable, multiscale platform for the modelling of tissue response to viral infections. The model was introduced using hepatitis C as an example and it was proposed that it could be adapted to other hosts and virus types.

In the present paper, we consider a specific experimental setting for which we aim at identifying a quantitative model that can be parametrized based on the collected data and which allows verifiable predictions. This requires a compromise between model complexity and considered model features for which quantitative information is available. In particular, we want to demonstrate how single-cell RNA sequencing (scRNA-Seq) data as a novel important data resource for systems-medicine applications can be utilized to establish such a quantitative model.

Several studies used Syrian hamsters as animal models for COVID-19^24–29^. Trimpert et al. compare the susceptibilities of three dwarf hamster species^30^ showing that Roborowski hamsters expressed the highest severity while Syrian hamsters express moderate infections. Other species were also considered, e.g., rhesus macaques^31^, 3 different non-human primates^32^, ferrets^33,34^, mice^35,36^ or cats^37^.

To generate high-quality data for modelling, we performed SARS-CoV-2 infection experiments and collected viral and cellular readouts at five time points. In particular, we applied scRNA-Seq to identify the dynamics of relevant cell populations at the site of infection. Data are used for model parametrization and validation. Our proposed model describes dynamics of the virus, alveolar epithelial cells, and cells and humoral factors of the innate and adaptive immune response to SARS-CoV-2 including important cytokine signalling feedbacks, where we pay particular attention to the early times post infection.

## Results

We developed a biomathematical model of Sars-CoV-2 infection in Syrian hamsters based on mechanistic assumptions about the interplay between virus, epithelial cells, immune cells and humoral factors of the immune response including different types of chemokines and antibodies. Assumptions are explained and discussed in detail in the methods section. There, we also derive the model equations.

**Fig. 1:**
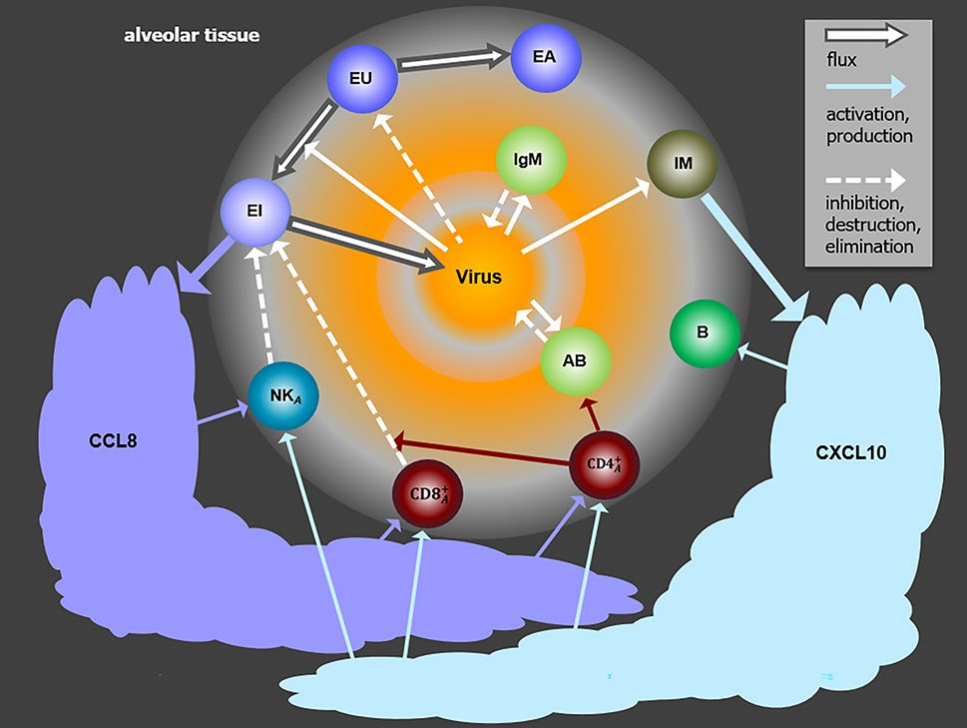
Structure of the model. The model describes the dynamics of epithelial cells (EU), activated epithelial cells (EA), infected epithelial cells (EI), activated ${\text {CD4}^+}$ T cells (${\text {CD4}^+_A}$), activated ${\text {CD8}^+}$ T cells (${\text {CD8}^+_A}$), monocyte-derived macrophages in lungs (IM), activated natural killer cells ($\text {NK}_A$), neutralising antibodies of IgM type (IgM), neutralizing antibodies (AB), B cells (B), the chemokines C-C motif ligand 8 (CCL8), and C-X-C motif ligand 10 (CXCL10), and viral load (V). Type and direction of arrows indicate cell fluxes or actions.

Major cell and cytokine compartments, feedbacks and actions of our model are presented in Fig. 1. Model components and their biological counterparts are summarized in Table 1.

**Table 1:** Model compartments and data.

Model component	Biological meaning	Corresponding experimental readouts
EU	Unaffected alveolar epithelial	–
	Cells type 2 (AT2)	
EA	Activated AT2 cells	–
EI	Infected AT2 cells	SARS-CoV-2 positive AT2 cells per lung lobe
IM	Inflammatory macrophages	Monocyte derived macrophages per lung lobe
${\text {CD8}^+_A}$	Activated $\text {CD8}^+$ T cells	*Gzma* positive $\text {CD8}^+$ T cells per lung lobe
${\text {CD4}^+_A}$	Activated $\text {CD4}^+$ T cells	*Ifng* positive $\text {CD4}^+$ T cells per lung lobe
${\text {NK}_A}$	Activated natural killer cells	*Ifng* positive natural killer cells per lung lobe
IgM	Antibodies of IgM type	Protein expression values for IgM heavy chains in lung
AB	Neutralizing antibodies	Serum neutralization titers
B	B cells	B cells per lung lobe
CCL8	Chemokine CCL8	No direct data available, production assumed to be proportional to gene-expression of AT2 cells (scRNAseq)
CXCL10	CXC chemokine ligand 10 (CXCL10)	No direct data available, production assumed to be proportional to gene-expression of macrophages (scRNAseq)
V	Virus	Virus titers in lung homogenates

We present major model compartments and respective available readouts from animal experiments. GzmA = Granzyme A, *Gzma* positive cells = cells expressing Granzyme A, IgM = Immunglobulin M, IFN$\gamma$ = Interferon gamma, *Ifng* positive cells = cells expressing interferon gamma

### Parameter estimates

Unknown parameters of the model were estimated to optimize the agreement of model predictions and experimental data. Data were collected at days 2, 3, 5, and 14 after infection and comprise viral loads, cell counts of $\text {CD4}^+$ T cells, $\text {CD8}^+$ T cells, macrophages, natural killer cells and B cells, as well as antibodies. Most importantly, lung homogenates were subjected to scRNA-sequencing analysis to determine immunce-cell fractions and their expression profiles. Readouts of the model and corresponding experimental data are shown in Table 1.

Estimated parameter values and initial values are presented in Tables S1-S2 in the supplement material (S1_File). For model calculations, we considered dimensionless parameters. We use steady state values of “1” (or zero) for the model compartments. For the sake of comparisons with the data, model output is multiplied by the mean of respective measurements at time zero.

**Fig. 2:**
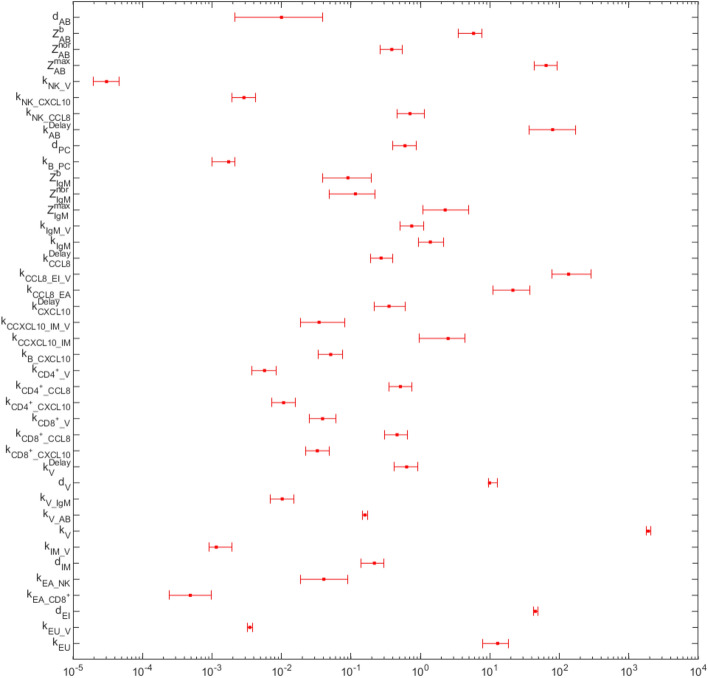
Estimates and confidence intervals of model parameters. We show parameters and corresponding 95 % confidence intervals derived from simulation. The parameter $d_{AB}$ could not be estimated with a high accuracy, and therefore, was set to 0.01.

Confidence intervals of parameter estimates are determined with bootstrapping. We created virtual random data points using the empirical distribution of measurements and fitted parameters to these virtual data. Resulting distributions of parameter values are used to define empirical confidence intervals (see Fig. 2, see methods for details). Since data are retrieved within the first two weeks of infection, specific antibody waning $d_\text {AB}$ could not be estimated with high precision, and the confidence interval is very large. Therefore, we decided to set the parameter to 0.01 and to study corresponding model behaviour in the results section. Confidence intervals of parameters $k_{EA\_NK}$ and $k_{EA\_CD8^{+}}$ are also large because these parameters could compensate each other to some extent.

Results of sensitivity analysis are shown in S1_File, Figure S1. Parameters showed a reasonable identifiability (Figure S1). Parameters affecting growth, spread and degradation of virus or target cells are particularly sensitive. This comprises the parameters susceptible cells infection rate ${\textit{k}}_{\text {EU}\_\text {V}}$, infected cells removal rate ${\textit{d}}_{\text {EI}}$, free virus degradation ${\textit{d}}_{\text {V}}$, and virus growth rate ${\textit{k}}_{\text {V}}$.

Finally, we analysed the correlations between the parameter estimates. As expected, a few parameter estimates are correlated because mechanisms acting in opposite directions can compensate each other to some extent. Estimates of correlations are displayed in Figure S2. The median of the absolute values of the correlations was 0.072 and the interquartile range was 0.030–0.14. Stronger correlations were observed for only four of our parameters, namely $d_{EI}$, $d_V$, $k_{EU\_V}$ and $k_V$, all relevant for the description of virus dynamics. Thus, it is possible to replace these parameter estimates by suitable literature findings if available. Correlations of all other parameters were low to moderate ensuring good identifiability.

### General model behaviour

To analyse the general behaviour of our model, we simulate its dynamics over 100 days after infection. In this time frame, all model components return to normal values except for the specific antibodies, which continuously decrease but remain elevated until the end of the simulated period.

**Fig. 3:**
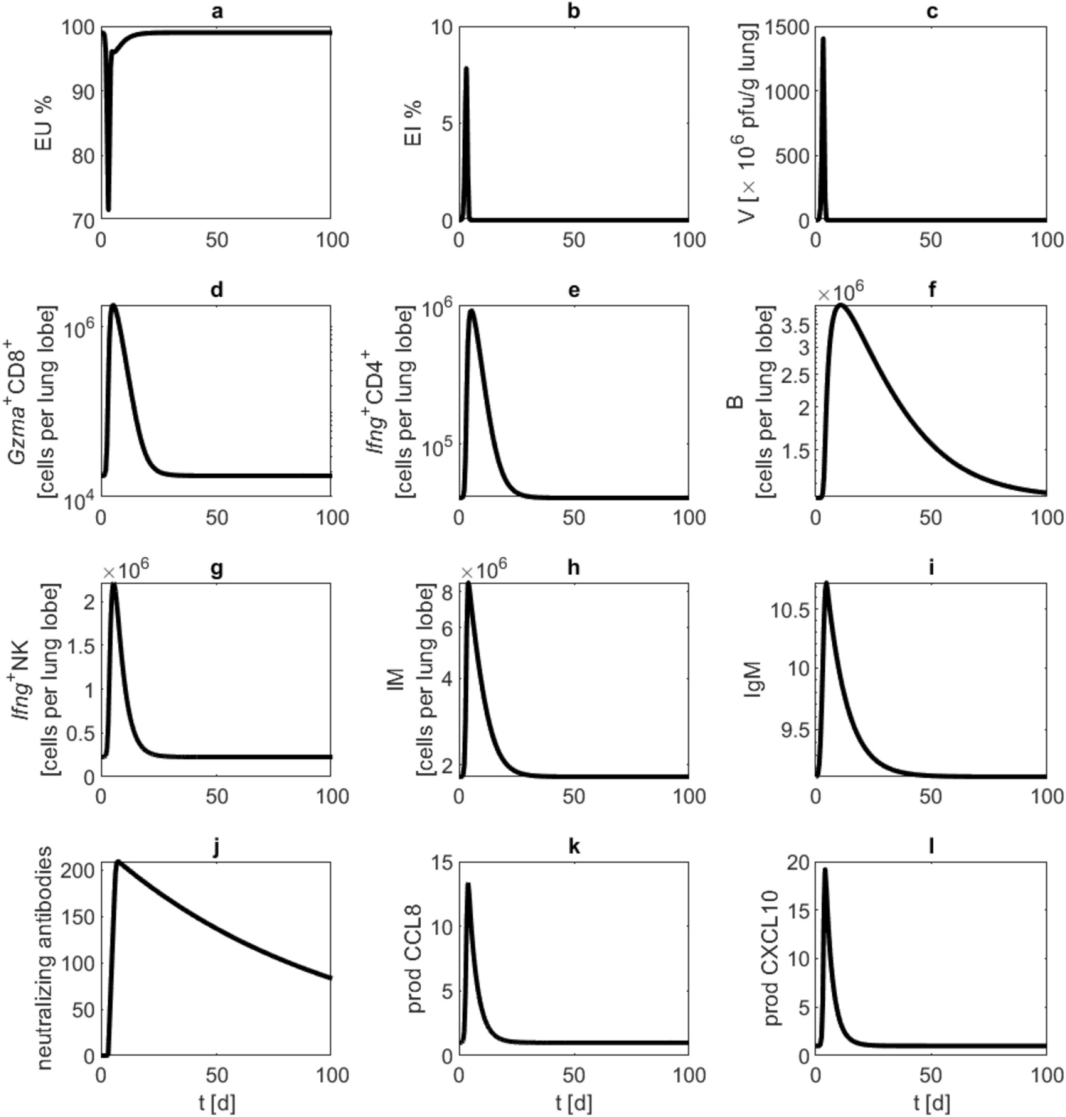
Long-term behaviour. We present results of long term simulation (100 days) of our model of moderate SARS-CoV-2 in Syrian hamsters. Black curves represent model simulations of (**a**) unaffected epithelial cells, (**b**) infected epithelial cells, (**c**) virus load, (**d**) activated $\text {CD8}^+$ T cells, (**e**) activated $\text {CD4}^+$ T cells, (**f**) B cells, (**g**) activated NK cells, (**h**) monocyte derived macrophages, (**i**) antibodies of IgM type, (**j**) neutralizing antibodies, (**k**) CCL8 production by activated epithelial cells, (**l**) CXCL10 production by macrophages.

While the virus is removed after about five days, cell counts of activated $\text {CD8}^+$ T cells, $\text {CD4}^+$ T cells, monocyte derived macrophages and NK cells return to normal levels within about three to six weeks after infection. The numbers of B cells and antibodies remain elevated for a longer time. With our parameter setting, after about 10 to 12 weeks, 50% of neutralising antibodies are still present (see Fig. 3). However, it needs to be acknowledged that the specific antibody waning rate $d_{AB}$ could not be estimated with sufficient accuracy since extrapolation is difficult and longer data time series were not available.

### Comparison of model and data

**Fig. 4:**
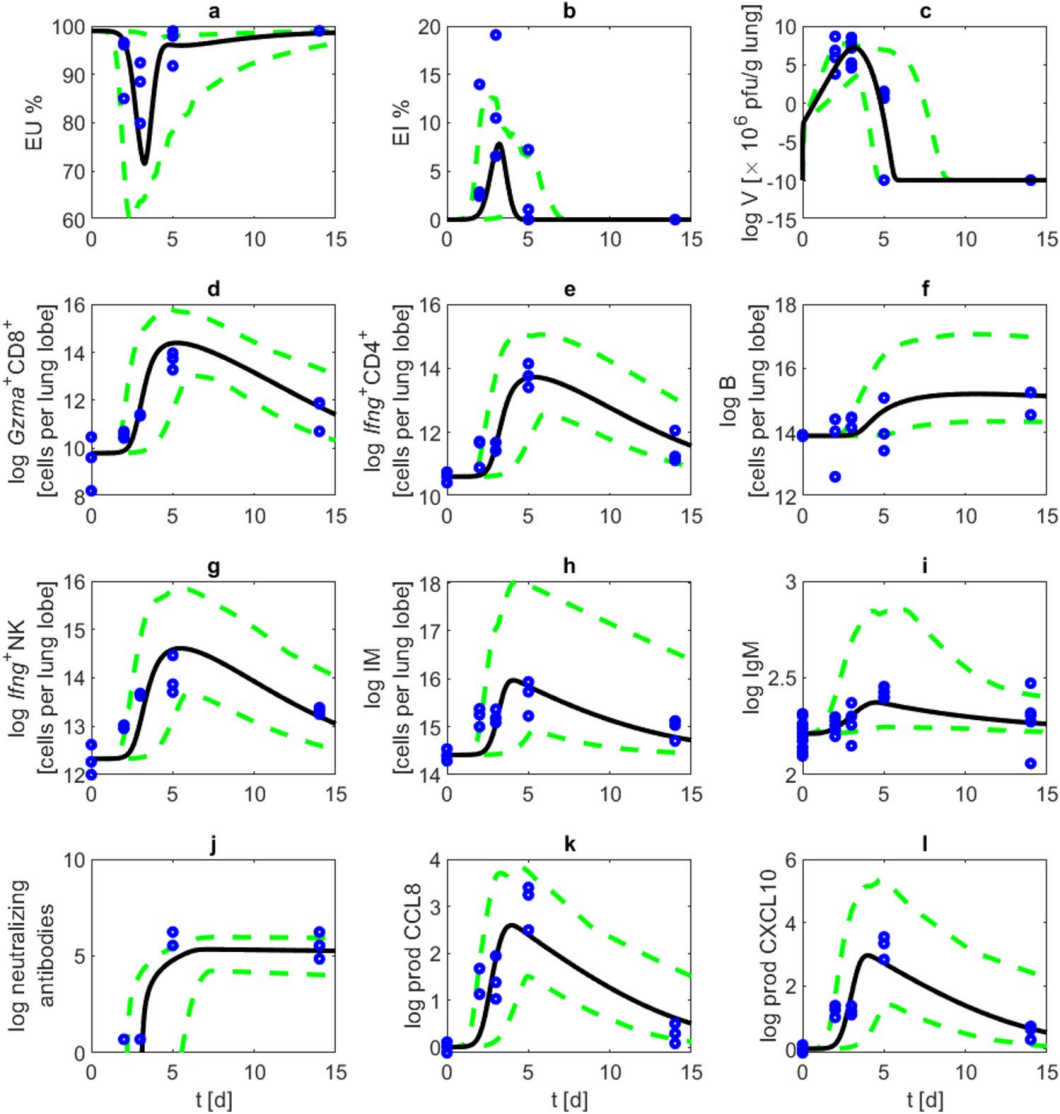
Model and data. We compare simulations of our model with available data. Blue: data points, red: mean and standard deviation of experimental data. Black curves represent model simulations of (**a**) unaffected epithelial cells, (**b**) infected epithelial cells, (**c**) virus load, (**d**) activated $\text {CD8}^+$ T cells, (**e**) activated $\text {CD4}^+$ T cells, (**f**) B cells, (**g**) activated NK cells, (**h**) monocyte derived macrophages, (**i**) antibodies of IgM type, (**j**) neutralizing antibodies, (**k**) CCL8 production by activated epithelial cells, (**l**) CXCL10 production by macrophages. Blue points show data, and 95% confidence bands of model predictions are displayed with green dashed lines.

Parameter estimates resulted in a good agreement of model and data (see Supplemental Table S3 for fitness values and information criteria, see also confidence bands of Figure 4). To compare model results and data, we use plaque-forming units (PFU) as a proxy for viral load. Starting the infection at day zero with $10^5$ PFU, maximal viral load is reached at days two and three post infection. Early infection markers are rising levels of IgM and monocyte derived macrophages with maximum concentrations already achieved at day four. Cell counts of activated $\text {CD4}^+$ T cells, $\text {CD8}^+$ T cells and NK cells reach their maximum values at about day five and six, and neutralising antibodies peak at about day seven and eight. About two weeks after infection, the maximum number of B cells is predicted (see Fig. 4).

### Model predictions

**Fig. 5:**
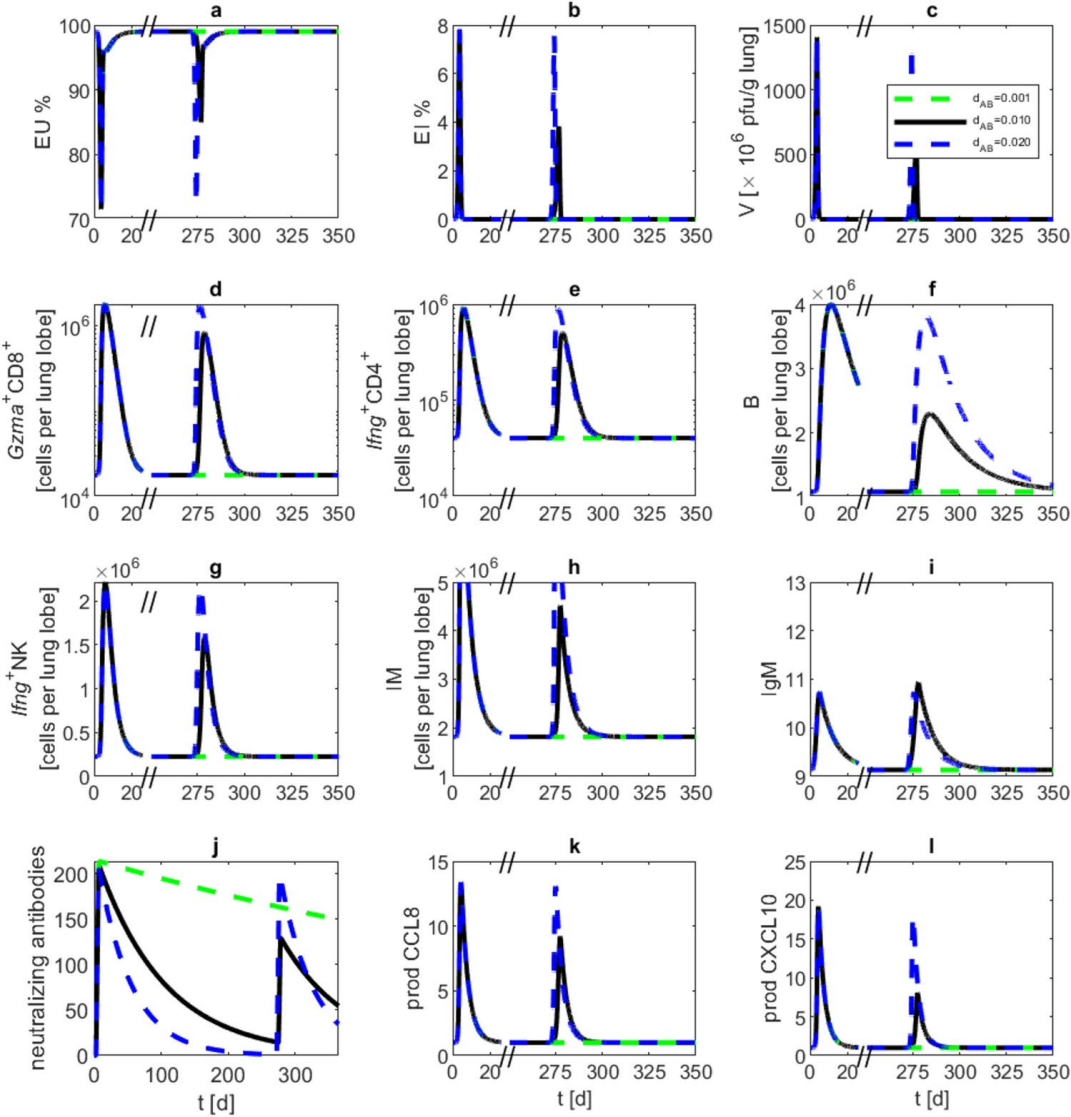
Reinfection attempt at day 270 after the first infection simulated with different parameter settings for antibody waning $d_{\text {AB}}$. We performed model simulations with a first infection event at day zero with $10^5$ pfu, and a reinfection event at day 270 with the same viral load. Lines (waning rates 0.001—green, 0.01—black and 0.02—blue) represent model simulations of (**a**) unaffected epithelial cells, (**b**) infected epithelial cells, (**c**) virus load, (**d**) activated $\text {CD8}^+$ T cells, (**e**) activated $\text {CD4}^+$ T cells, (**f**) B cells, (**g**) activated NK cells, (**h**) monocyte derived macrophages, (**i**) antibodies of IgM type, (**j**) neutralizing antibodies, (**k**) CCL8 production by activated epithelial cells, (**l**) CXCL10 production by macrophages. Lower waning rates ameliorate or even prevent the secondary infection event.

Since the antibody waning parameter $d_{AB}$ could not be estimated with sufficient accuracy, we simulated reinfection scenario for different parameter settings providing predictions verifiable in future animal studies. Initial infection starts at day zero with $10^5$ pfu, and a reinfection attempt with the same viral load was modelled at day 270. Waning rates are set to 0.001, 0.01 and 0.02, respectively. With a small waning rate of 0.001, antibody concentration is sufficiently high to prevent the reinfection. With waning rates of 0.01 and 0.02, we see an increasing immune response to the second infection attempt (see Fig. 5).

**Fig. 6:**
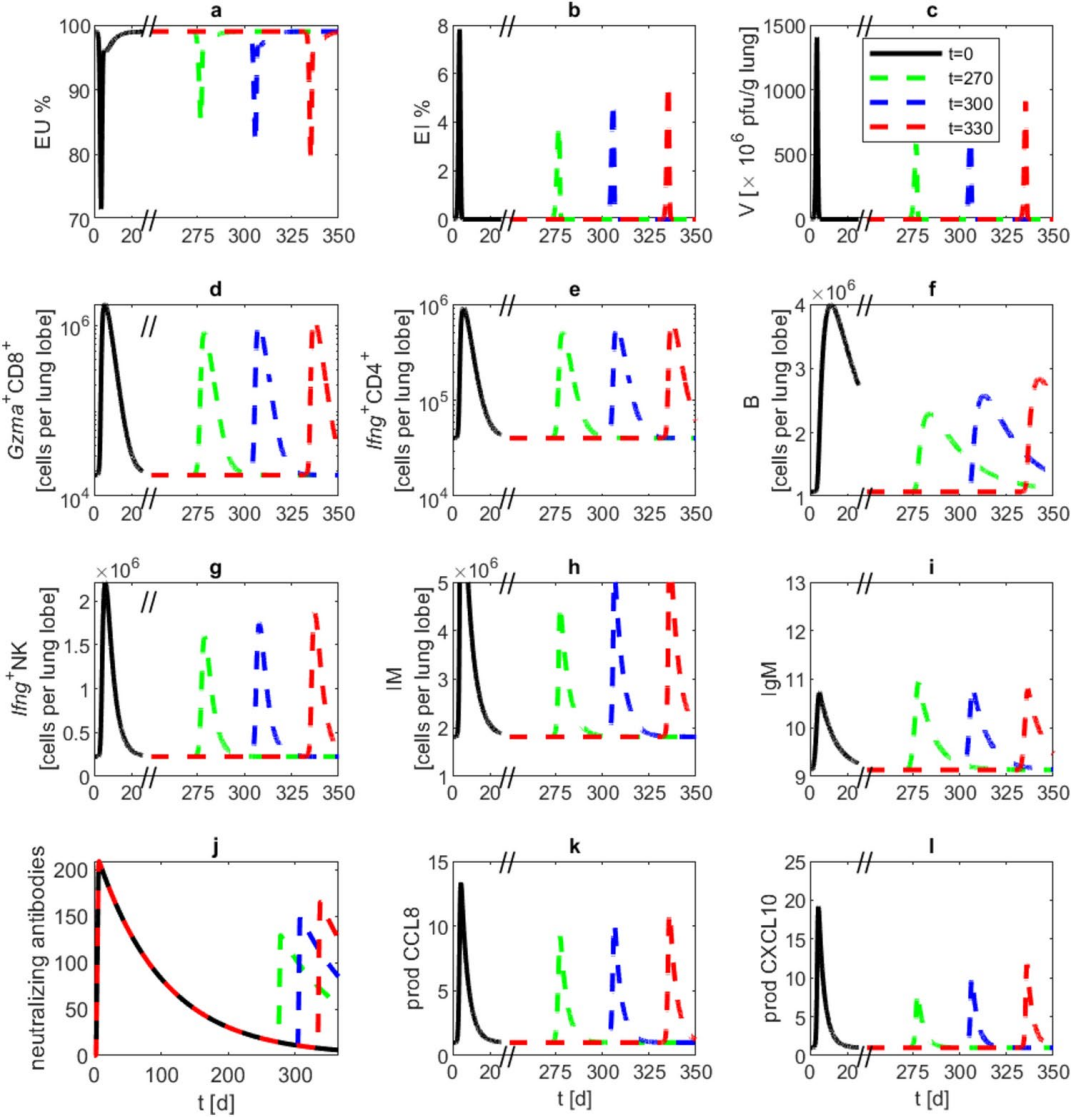
Reinfection at different time points of antibody waning. We present model simulations of a second infection event at different time points after the first infection event at day zero with $10^5$ pfu and $d_{\text {AB}}=0.1$. Reinfection events were modelled at days 270 (green), 300 (blue), and 330 (red), again with $10^5$ pfu. Black lines correspond to a simulation without reinfection. Lines represent model simulations of (**a**) unaffected epithelial cells, (**b**) infected epithelial cells, (**c**) virus load, (**d**) activated $\text {CD8}^+$ T cells, (**e**) activated $\text {CD4}^+$ T cells, (**f**) B cells, (**g**) activated NK cells, (**h**) monocyte derived macrophages, (**i**) antibodies of IgM type, (**j**) neutralizing antibodies, (**k**) CCL8 production by activated epithelial cells, (**l**) CXCL10 production by macrophages.

In another simulation analysis, we varied the time point of reinfection attempts to study the resulting immune response assuming an antibody waning of $d_{\text {AB}}=0.1$. For this purpose, we re-challenged our model with the same virus load, at 270, 300, or 330 days after first infection. In all simulations, immune response was reduced compared to the first infection. Peaks of activated T and NK cells, B cells, monocyte derived macrophages, CCL8—and CXCL10 production are clearly lower than at the initial infection event. This is mainly due to the neutralising antibodies remaining elevated for a longer time period, and with it, diminishing overall immune response. Accordingly, a trend towards higher immune response maxima was observed for later occuring secondary infection events Fig. 6.

**Fig. 7:**
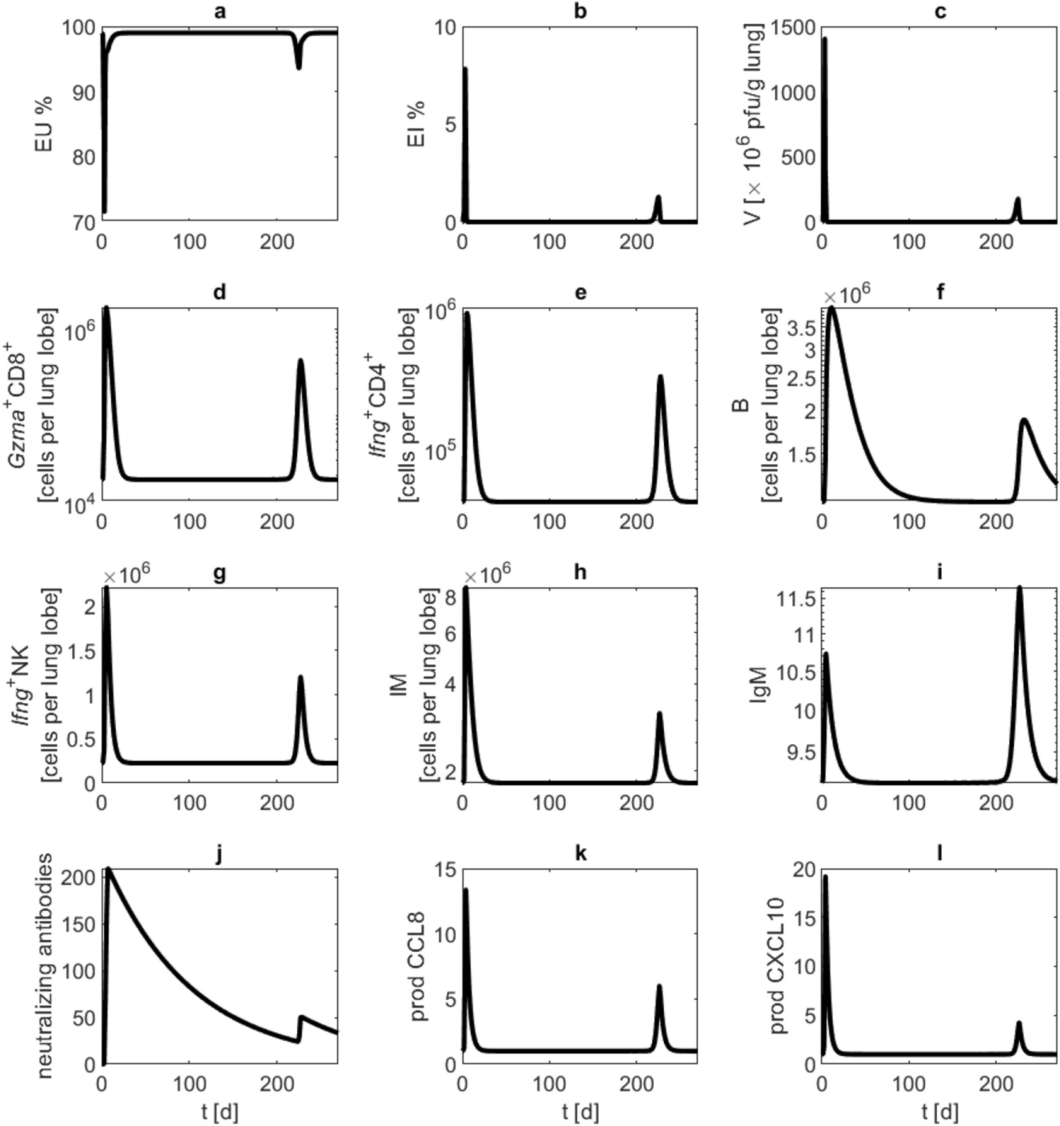
Repeated contact to the virus. We present a model simulation of repeated infections with $10^5$ pfu at day zero, and after 50 days repeatedly every 10 days, antibody waning $d_{\text {AB}}$ is set to 0.1. Black curves represent model simulations of (**a**) unaffected epithelial cells, (**b**) infected epithelial cells, (**c**) virus load, (**d**) activated $\text {CD8}^+$ T cells, (**e**) activated $\text {CD4}^+$ T cells, (**f**) B cells, (**g**) activated NK cells, (**h**) monocyte derived macrophages, (**i**) antibodies of IgM type, (**j**) neutralizing antibodies, (**k**) CCL8 production by activated epithelial cells, (**l**) CXCL10 production by macrophages. Immune response is predicted to occur only occasionally if neutralising antibodies drop below a critical level.

Finally, we simulated repetitive reinfection events. For this purpose, we assumed infections with $10^5$ pfu at day 0 and every 10 days after day 50. Antibody waning $d_{\text {AB}}$ is set to 0.1. Of note, a larger immune response is only evoked at time point 200 at which antibodies dropped below a critical limit of about 30, which is about 7% from its maximum value (Fig. 7).

## Discussion

Understanding the mechanisms of SARS-CoV-2 infection and induced immune responses is important for the development of improved and individualized therapies of COVID-19. We here proposed a model of SARS-CoV-2 in Syrian hamsters mirroring mild to moderate COVID-19. Our model was parametrized on the basis of our own experimental data obtained from SARS-CoV-2 or mock infected animals. Model parametrization resulted in a good agreement of model predictions and data and a well-identifiable set of parameters. We demonstrated how the model can be used to provide verifiable predictions e.g., of repeated infections.

We used a dataset of SARS-CoV-2 infection of Syrian hamsters to have a sufficiently rich data base for our modelling efforts. In particular, with the help of single-cell analyses, we established data of the dynamics of major immune cell populations and their chemokine expressions relevant to understand the intercellular communications between major players of the immune response. To our knowledge, this is the first model of SARS-CoV-2 infection in hamsters using single-cell data as primary learning resource. Although there are other biomathematical models of SARS-CoV-2 infections proposed in the literature, these models were developed for other species, other areas of applications or are based on other data resources so that translation of these models to our situation is not straight-forward.

In our modelling framework, we included major physiological players of the immune response and their interactions including virus population, affected alveolar cells, activated T cells, macrophages, activated natural killer cells and B cells, neutralising antibodies of IgM type, neutralizing antibodies, and the chemokines CCL8 and CXCL10. Selection of major players and mechanisms was based on proposed major (patho-)physiological processes and literature data.

We refrained from considering differentiation of subtypes of $\text {CD4}^+$ T cells such as Th1, Tfh, CD4 T and CD4-CTL^14,38^. This differentiation is more likely to take place in the lymph nodes and respective data were not available in our experimental setting. In our data, dendritic cells showed no significant dynamics. Thus, we decided to neglect them in our model. CXCL10 and CCL8 transcripts displayed the strongest increase in pulmonary expression levels in response to SARS-CoV-2 infection^39^. They are also known to be intensively involved in the recruitment of immune cells. Therefore, we included these two chemokines in the model. To avoid increasing the complexity of the model, we do not include further cytokines.

Due to short time series of our data, specific antibody waning parameters could not be identified. We therefore performed simulations assuming different values of this parameter demonstrating that it considerably affects dynamics after re-infections. Longer time series or repeated infection challenges of the system are required to assess antibody waning and effectiveness and to improve our model in this regard. As a notable finding, we predict that there is a critical limit of specific antibodies evoking an immune response after re-infections while higher values immediately stop the infection. Further experiments are required to validate our long-term predictions and to improve our model. In principle, our model can also be used to perform simulations of short-term interventions such as medication. This, however, would likely require additional model assumptions regarding drug pharmacokinetics and -dynamics.

In our model, we only considered a single variant, namely ancestral SARS-CoV-2 variant B.1 (SARS-CoV-2 isolate BetaCoV/Germany/BavPat1/2020). Generalization of our model to other variants is not straightforward. But, it is likely that this can be achieved by adjusting a limited set of model parameters. We are also interested in studying more severe disease conditions of COVID-19 as in^14^. This would require considering other animal models such as the Roborovski dwarf hamster^30^.

We conclude that we established a comprehensive biomathematical model of SARS-CoV-2 in Syrian hamsters. The selected immunologic mechanisms considered in our model proved to be sufficient to explain the data of our experiments also allowing estimating a set of physiological parameters. We aim to validate our model on the basis of future experimental data. We also plan to translate the model to more severe disease conditions and to include therapy options such as Nirmatrelvir/Ritonavir.

## Methods

In the following, we present and briefly discuss our model hypotheses required to derive the model equations.

### Model assumptions and equations

The basic model structure is based on the following mechanisms. After infection, type 2 alveolar epithelial cells (AT2) are targeted by the virus^40^. Infection of AT2 cells results in virus production and CCL8 release. Activated T cells and natural killer (NK) cells are attracted by CCL8 and destroy virus-infected epithelial cells^41^. The presence of viruses is assumed to activate further T—and NK cells^42–45^. Virus sensing or phagocytosis of viruses by macrophages results in CXCL10 production. In addition to attracting activated T and NK cells, CXCL10 recruits B cells^46^, which can differentiate into IgM secreting plasma cells when encountering their cognate antigen. With the help of $\text {CD4}^+$ T cells, B cells undergo class switch reactions, and affinity maturation, thus differentiate into plasma cells producing antibodies neutralizing free viruses^6^. Based on these assumptions, we derive our model equations in the following. Dimensionless parameters are used because most of our data are semi-quantitative.

#### Virus dynamics

Infection is initiated by aspirating a certain amount of virus particles modelled as an injection function $\text {V}_\text {start}$. In our experiments, we applied an initial dose of $10^5$ infectious virus particles intranasally, which infect epithelial cells *EI*. The dose of $10^5$ plaque-forming units (pfu) of SARS-CoV-2 was already used in other studies^25–27,29,30^. A lower dose of 5,000 pfu of SARS-CoV-2 was tested in dwarf hamsters^30^. Infected cells produce new virus particles with replication rate $k_\text {V}$. Free viruses are cleared with rate $d_\text {V}$ or neutralized by IgM-type and neutralising antibodies at rates $k_\text {V}\_\text {IgM}$ and $k_\text {V}\_\text {AB}$, respectively.

1 $$\begin{aligned} \frac{d \text {V}}{dt}=&k_\text {V}\cdot \text {EI} - ( k_\text {V}\_\text {AB} \cdot \text {AB} + k_\text {V}\_\text {IgM} \cdot \text {IgM} + d_\text {V} ) \cdot \text {V} + \text {V}_\text {start} \end{aligned}$$

with

2 $$\begin{aligned} \text {V}_\text {start} = \sum _{i=1}^{N} \frac{ {\mathrm {dose\_V}}_{i}}{t_{\textrm{V}}} \big (\textrm{Hv}(t-\tilde{t_i})-\textrm{Hv}(t-\tilde{t_i}- {t_{\textrm{V}}})\big ) \end{aligned}$$

where Hv is the Heaviside-function $$\textrm{Hv}=\left\{ \begin{array}{r@{\quad : \quad }l} 0 & x<0 \\ 1 & x\ge 0 \end{array} \right.$$, $\tilde{t_i}$ are the time points at which viruses at dose $\mathrm {dose\_V}_i$ are administered within time $t_{\textrm{V}}$. Equation (2) results in function which is constant and non-zero between the time points $\tilde{t_i}$ and $\tilde{t_i}+t_{\textrm{V}}$, where $t_{\textrm{V}}$ is the application time of the virus. The function is zero outside of application windows. The injection dose $\mathrm {dose\_V}_i$ corresponds to the area under the curve of the given application window. This injection function is convenient to simulate multiple infection events. The value of $\mathrm {dose\_V}_i$ was set to 0.1 [$\times 10^6$ pfu], and $t_{\textrm{V}}$ was set to 6 min. The injection time is virtually irrelevant for model dynamics and was set, i.e. no additional parameter is introduced by our injection function. The part of viruses which is cleared by macrophage phagocytosis is assumed to be very small in comparison to the neutralisation by antibodies and therefore neglected in Eq. 1.

#### Epithelial cells

In our model, we distinguish between unaffected (EU), SARS-CoV-2 infected (EI) and activated epithelial cells without SARS-CoV-2 infection (EA). Unaffected type 2 alveolar epithelial cells (EU) are targeted by SARS-CoV-2^47^, become infected and produce new viruses. Respective transition of EU towards EI is modelled proportional to contacts of EU and V. Epithelial cells can be activated by pathogens, cytokines or other impairments, and in response, produce several cytokines^39^. Similar to^48,49^, we normalize AT2 cells to 100 %, and we assume that 1% of AT2 cells are activated in equilibrium, caused by general stress from interferon response and apoptosis. We here assume a constant transition of EU towards EA with rate $k_{EU}$. Loss of epithelial cells is caused by damage induced by immune cells invading the alveolus and is assumed proportional to ${\text {NK}_A}$ and ${\text {CD8}^+_A}$ cell counts^38,50^. Moreover, ${\text {CD8}^+_A}$ T cells actively attack infected cells with the help of ${\text {CD4}^+_A}$ T cells. We also assume an unspecific loss of cells from EI and EA with rates $d_\text {EI}$ respectively $d_\text {EA}$. In our model, there is no cell flux from EA to EU, i.e. EA cells are not infectable.

3 $$\begin{aligned} \frac{d \text {EU}}{dt}&= P_\text {EU} - k_\text {EU}\_\text {V} \cdot \text {EU} \cdot \text {V} -k_{\text {EA}\_\text {CD8}^+} \cdot \text {EU} \cdot {\text {CD8}^+_A} -k_\text {EA}\_\text {NK} \cdot \text {EU} \cdot {\text {NK}_A} -k_\text {EU} \cdot \text {EU} \end{aligned}$$

4 $$\begin{aligned} P_\text {EU}&= k_{\text {EA}\_\text {CD8}^+} \cdot {\text {CD8}^+_A}_0 \cdot \text {EU}_0 + k_\text {EA}\_\text {NK} \cdot {\text {NK}_A}_0 \cdot \text {EU}_0 + k_\text {EU} \cdot \text {EU}_0 \nonumber \\ \frac{d \text {EI}}{dt}&= k_\text {EU}\_\text {V} \cdot \text {EU} \cdot \text {V} -k_{\text {EA}\_\text {CD8}^+} \cdot \text {EI} \cdot {\text {CD8}^+_A} \cdot (1+{\text {CD4}^+_A} )-k_\text {EA}\_\text {NK} \cdot \text {EI} \cdot {\text {NK}_A}\nonumber \\&\quad -d_\text {EI} \cdot \text {EI} \end{aligned}$$

5 $$\begin{aligned} \frac{d \text {EA}}{dt}&= k_\text {EU} \cdot \text {EU} - k_{\text {EA}\_\text {CD8}^+} \cdot \text {EA} \cdot {\text {CD8}^+_A} - k_\text {EA}\_\text {NK} \cdot \text {EA} \cdot {\text {NK}_A} - d_\text {EA} \cdot \text {EA} \nonumber \\ d_\text {EA}&= \left( k_{EU} \cdot \text {EU}_0 - k_{\text {EA}\_\text {CD8}^+} \cdot \text {EA}_0 \cdot {\text {CD8}^+_A}_0 - k_{\text {EA}\_\text {NK}} \cdot {\text {NK}_A}_0 \cdot \text {EA}_0 \right) / \text {EA}_0 \end{aligned}$$

where $P_\text {EU}$ represents production of EU balancing cell loss in steady-state. Likewise, $d_\text {EA}$ is constructed in such a way that a constant steady-state is established. The index ”0” always represents steady-state values in the following equations.

#### Inflammatory macrophages in the lung

Pulmonary monocyte-derived macrophages (IM) play a key role in immune defense. Usually, monocytes and macrophages are activated by cytokines. According to our data^39^, infected epithelial cells show low cytokine release, while macrophages early and strongly respond to viral RNA by producing pro-inflammatory cytokines. Therefore, we assume that activation of macrophages occurs through contact with the virus itself. Detection of viral RNA in monocytes and macrophages supports this hypothesis^39,51–53^.

6 $$\begin{aligned} \frac{d \text {IM}}{dt}&= d_\text {IM}\cdot \left( \text {IM}_0 - \text {IM} \right) + k_{\text {IM}\_\text {V}} \cdot \text {IM} \cdot \text {V} \end{aligned}$$

where $k_{\text {IM}\_\text {V}} \cdot \text {IM} \cdot \text {V}$ is the recruitment rate of IM due to virus attack, $d_\text {IM}$ represents the natural decay and baseline production rate of IM to ensure a constant steady-state $\text {IM}_0=1$. For comparison of model and data, model output of IM is multiplied by the observed value of inflammatory macrophages in control group.

#### Activated natural killer cells

Natural killer cells, as part of the innate immune system, are recruited early upon infection. NK cells have the task of eliminating infected cells limiting the viral spread^50^. We assume recruitment of activated NK cells (NK) by CXCL10^54,55^ and CCL8^56^ with rates $k_{{\text {NK}}\_\text {CXCL10}}$ and $k_{{\text {NK}}\_\text {CCL8}}$ respectively. Several additional activators of NK cells are known, e.g., type 1 interferons, IL-2, IL-12 and IL-15, which are produced by a variety of cell types in reaction to viral activity^42–44^. This complex network cannot be modelled here. In our expression data in the lungs, CXCL10 and CCL8 show comparatively strong dynamics^39^, hence we decided to include these two chemokines in the model. For the sake of simplicity, we further assume that the presence of viral RNA directly activates NK cells. This is motivated by the correlation of gene sets related to response to interferon-gamma with the presence of viral RNA^39^. We include it in our model via an additional virus-induced activation rate $k_{{\text {NK}}\_\text {V}}$ of NK cells, which were also recruited by CCL8 and CXCL10, and contribute to the (activated) NK compartment ${\text {NK}_A}$ fighting virus-infected ${\text {EI}}$ cells.

7 $$\begin{aligned} \frac{d {\text {NK}_A}}{dt}&= k_{\text {NK}\_\text {CCL8}}\cdot \text {CCL8} + k_{\text {NK}\_\text {CXCL10}}\cdot \text {CXCL10} + k_{{\text {NK}}\_\text {V}}\cdot (k_{{\text {NK}}\_\text {CCL8}}\cdot \text {CCL8} +k_{{\text {NK}}\_\text {CXCL10}}\cdot \text {CXCL10} ) \cdot \text {V}\nonumber \\&\quad -d_\text {NK} \cdot {\text {NK}_A} \nonumber \\ d_\text {NK}&= (k_{\text {NK}\_\text {CCL8}}\cdot \text {CCL8}_0 + k_{\text {NK}\_\text {CXCL10}}\cdot \text {CXCL10}_0)/{\text {NK}_A}_0 \end{aligned}$$

Here, $d_\text {NK}$ denotes the natural decay rate. Compartment ${\text {NK}_A}$ is normalized by ${\text {NK}_A}_0=1$. To compare the simulated time course of activated NK cells with observations, model output of ${\text {NK}_A}$ is multiplied by mean of *Ifng* positive NK cells in control group. IFN$\gamma$ itself is not explicitly modelled.

#### T cells

T-cells activate innate effector cells, eliminate damaged cells and destroy pathogens by various mechanisms. We here consider the classes of T helper cells ($\text {CD4}^+$) and cytotoxic T cells $\text {CD8}^+$, while their subtypes are not distinguished for simplicity. We assume recruitment of activated T cells by CCL8^57,58^ and CXCL10^59,60^. Activated $\text {CD8}^+$ T cells express Granzyme A (GzmA), which can induce cell death in target cells^45^, and activated ${\text {CD4}^+}$ T cells express IFN$\gamma$. In our model, the compartment ${\text {CD8}^+_A}$ describes *Gzma* positive $\text {CD8}^+$ T cells, and ${\text {CD4}^+_A}$ contains *Ifng* positive $\text {CD4}^+$ T cells. The presence of viral RNA is assumed to activate GzmA expression respectively IFN$\gamma$ expression in T cells^45^. In analogy to the NK compartment, this additional activation is modelled by delayed virus-induced activation compartments $\text {CD8}^+_\text {ViA}$, respectively $\text {CD4}^+_\text {ViA}$, whose effluxes contribute to the respective T cell compartments.

8 $$\begin{aligned} \frac{d {\text {CD8}^+_A}}{dt}&= k_{{\text {CD8}^+}\_\text {CCL8}}\cdot \text {CCL8} +k_{{\text {CD8}^+}\_\text {CXCL10}}\cdot \text {CXCL10} +k^{\textrm{Delay}}_{\textrm{V}}\cdot \text {CD8}^+_\text {ViA} - d_{\text {CD8}^+} \cdot {\text {CD8}^+_A} \nonumber \\ d_{\text {CD8}^+}&= (k_{{\text {CD8}^+}\_\text {CCL8}}\cdot \text {CCL8}_0 + k_{{\text {CD8}^+}\_\text {CXCL10}}\cdot \text {CXCL10}_0)/ {\text {CD8}^+_A}_0 \end{aligned}$$

9 $$\begin{aligned} \frac{d \text {CD8}^+_\text {ViA}}{dt}&= k_{{\text {CD8}^+}\_\text {V}}\cdot (k_{{\text {CD8}^+}\_\text {CCL8}}\cdot \text {CCL8} +k_{{\text {CD8}^+}\_\text {CXCL10}}\cdot \text {CXCL10} ) \cdot \text {V} -k^{\textrm{Delay}}_{\textrm{V}}\cdot \text {CD8}^+_\text {ViA} \end{aligned}$$

10 $$\begin{aligned} \frac{d {\text {CD4}^+_A}}{dt}&= k_{{\text {CD4}^+}\_\text {CCL8}}\cdot \text {CCL8} +k_{{\text {CD4}^+}\_\text {CXCL10}}\cdot \text {CXCL10} +k^{\textrm{Delay}}_{\textrm{V}}\cdot \text {CD4}^+_\text {ViA} - d_{\text {CD4}^+} \cdot {\text {CD4}^+_A}\nonumber \\ d_{\text {CD4}^+}&= (k_{{\text {CD4}^+}\_\text {CCL8}}\cdot \text {CCL8}_0 + k_{{\text {CD4}^+}\_\text {CXCL10}}\cdot \text {CXCL10}_0)/ {\text {CD4}^+_A}_0 \end{aligned}$$

11 $$\begin{aligned} \frac{d \text {CD4}^+_\text {ViA}}{dt}&= k_{{\text {CD4}^+}\_\text {V}}\cdot (k_{{\text {CD4}^+}\_\text {CCL8}}\cdot \text {CCL8} +k_{{\text {CD4}^+}\_\text {CXCL10}}\cdot \text {CXCL10} ) \cdot \text {V} -k^{\textrm{Delay}}_{\textrm{V}}\cdot \text {CD4}^+_\text {ViA} \end{aligned}$$

Here, $d_{\text {CD8}^+}$ respectively $d_{\text {CD4}^+}$ denote respective natural decay rates. T cell compartments are normalized by steady state values ${\text {CD8}^+_A}_0=1$ and ${\text {CD4}^+_A}_0=1$. To compare model prediction of activated T cells and experimental results, we use the data of respective *Gzma* or *Ifng* positive T cells from single-cell analysis and multiply model output by the respective value observed in control group.

#### B cells

B cells differentiate into IgM producing plasma cells following antigen recognition by their receptors, and, with the help of $\text {CD4}^+$ cells, undergo affinity maturation resulting in neutralising antibodies. We assume that B cells are recruited into alveolar space by CXCL10^46^.

12 $$\begin{aligned} \frac{d \text {B}}{dt}&= k_{\text {B}\_\text {CXCL10}}\cdot \text {CXCL10} -d_\text {B} \cdot \text {B}\nonumber \\ d_\text {B}&= k_{\text {B}\_\text {CXCL10}}\cdot \text {CXCL10} /\text {B}_0 \end{aligned}$$

The efflux rate $d_\text {B}$ includes migration into secondary lymphatic organs to mature into plasma cells, natural decay and other processes, where the value of $d_\text {B}$ results from equilibrium conditions.

#### Antibodies of IgM type

In the early acute phase of infection, activation of plasma cells results in IgM type antibodies (IgM) production in blood and lymph fluid^15,61^. Here we assume increased production of IgM in the presence of the virus, modelled with rate $k_{\text {IgM}\_\text {V}}$. We also assume that the total IgM production is limited, realized in our model by a sigmoid function $Z_{\textrm{IgM}}$.

13 $$\begin{aligned} \frac{d \text {IgM}}{dt} =&Z_{\textrm{IgM}}^{\textrm{max}}-(Z_{\textrm{IgM}}^{\textrm{max}}-Z_{\textrm{IgM}}^{\textrm{min}})\cdot e^{\big [-\big (\ln \big (\frac{Z_{\textrm{IgM}}^{\textrm{max}}-Z_{\textrm{IgM}}^{\textrm{min}}}{Z_{\textrm{IgM}}^{\textrm{max}}-Z_{\textrm{IgM}}^{\textrm{nor}}}\big )\big ) \cdot {(k_{\text {IgM}} +k_{\text {IgM}\_\text {V}}\cdot \text {V})}^{Z_{\textrm{IgM}}^{\textrm{b}}}\big ]} -d_\text {IgM} \cdot \text {IgM} \end{aligned}$$

The decay rate $d_\text {IgM}$ is calculated from steady state condition.

#### Neutralizing antibodies

Signals from $\text {CD4}^+$ T cells promote maturation of B cells in secondary lymphatic organs into plasma cells^62^. Simplifying this complex mechanism, we assume in our model affinity maturation by introducing a delay compartment $C_{\textrm{AB}}$, depending on the presence of ${\text {CD4}^+_A}$ T cells and the virus. With rate $k^{\textrm{Delay}}_{\textrm{AB}}$, mature cells transfer into the plasma cell compartment PC. This happens primarily in the germinal center reaction in secondary lymphoid organs, such as spleen and lymph nodes, and, only to a very small extent, at sites of infection when eventually tertiary lymphoid structures such as bronchus-associated lymphoid tissues are formed. Therefore, we refrain from including B cells in alveolar space into the equation of antibody formation.

14 $$\begin{aligned} \frac{d C_{\textrm{AB}}}{dt}&= k_{\text {B}\_\text {PC}} \cdot {\text {CD4}^+_A} \cdot \text {V} - k^{\textrm{Delay}}_{\textrm{AB}} \cdot C_{\textrm{AB}} \end{aligned}$$

15 $$\begin{aligned} \frac{d {\textrm{PC}}}{dt}&= k^{\textrm{Delay}}_{\textrm{AB}} \cdot C_{\textrm{AB}}-d_\text {PC}\cdot \text {PC} \end{aligned}$$

The compartment PC contains mature plasma cells, which produce neutralizing antibodies AB. $d_\text {PC}$ denotes the degradation rate of plasma cells. Production capacity is limited by a sigmoid function $Z_{\textrm{AB}}$.

16 $$\begin{aligned} \frac{d \text {AB}}{dt}&= Z_{\textrm{AB}}^{\textrm{max}}-(Z_{\textrm{AB}}^{\textrm{max}}-Z_{\textrm{AB}}^{\textrm{min}}) \cdot e^{\big [-\big (\ln \big (\frac{Z_{\textrm{AB}}^{\textrm{max}}-Z_{\textrm{AB}}^{\textrm{min}}}{Z_{\textrm{AB}}^{\textrm{max}}-Z_{\textrm{AB}}^{\textrm{nor}}}\big )\big ) \cdot {( \text {PC}) }^{Z_{\textrm{AB}}^{\textrm{b}}}\big ]} - d_\text {AB}\cdot \text {AB} \end{aligned}$$

Here, $d_\text {AB}$ denotes the decay rate of neutralizing antibodies.

#### Chemokine CCL8 (MCP-2)

The chemokine CCL8 (Monocyte Chemoattractant Protein 2 (MCP-2)) is involved in inflammatory processes and recruits various immune cells, e.g., T cells or NK cells^56–58^. CCL8 shows strong changes in the expression data in the lungs^39^. Not only the infected *EI* cells alone produce CCL8, but also cells surrounding *EI*, especially endothelial cells, are activated and participate in the production. This process is not considered in our model, for simplicity, we assume a delayed additional production by *EI* only. This is modelled by a delay compartment. Furthermore, we assume secretion of CCL8 by activated alveolar cells EA^39^.

17 $$\begin{aligned} \frac{d C_{\textrm{CCL8}}^{(1)}}{dt}&= k_{\text {CCL8}\_\text {EI}\_\text {V}}\cdot \text {EI} - k^{\textrm{Delay}}_{\textrm{CCL8}} \cdot C_{\textrm{CCL8}}^{(1)} \nonumber \\ C_{\mathrm {CCL8\_out}}^{(1)}&= k^{\textrm{Delay}}_{\textrm{CCL8}} \cdot C_{\textrm{CCL8}}^{(1)} \nonumber \\ \frac{d \text {CCL8}}{dt}&= k_{\text {CCL8}\_\text {EA}} \cdot \text {EA} +C_{\mathrm {CCL8\_out}}^{(1)} -d_\text {CCL8} \cdot \text {CCL8}\nonumber \\ d_\text {CCL8}&= \frac{k_{\text {CCL8}\_\text {EA}}\cdot \text {EA}_0}{\text {CCL8}_0} \end{aligned}$$

Due to insufficient availability of antibodies in hamsters, ELISA or FACS are not possible. Outside of proteomics, no protein measurement was carried out. From our single-cell data, we retrieved normalized CCL8 gene expressions of alveolar epithelial cells type 2. This gene-expression was related to the modelled relative CCL8 production as described by the following equation.

18 $$\begin{aligned} \text {CCL8}_\text {prod} = \frac{k_{\text {CCL8}\_\text {EA}}\cdot \text {EA}+C_{\mathrm {CCL8\_out}}^{(1)}}{k_{\text {CCL8}\_\text {EA}}\cdot \text {EA}_0} \end{aligned}$$

#### CXC chemokine ligand 10 (CXCL10, IP-10)

Chemokine CXCL10 is produced by a wide spectrum of cell types^54^. CXCL10 shows strong changes in the expression data in the lungs and can serve as a marker for pulmonary inflammatory processes^39^ and attracts different immune cells, e.g., NK cells^54^ or B cells^46^. In our model, CXCL10 is only produced by $\text {IM}$. The production is increased due to virus contacts (including phagocytosis^63^) with some delay. The delay is again modelled by a compartment. Epithelial cells are assumed irrelevant for CXCL10 production due to a weak reaction of these target cells as described in^39^.

19 $$\begin{aligned} \frac{d C_{\textrm{CXCL10}}^{(1)}}{dt}&= k_{\text {CXCL10}\_\text {IM}\_\text {V}}\cdot \text {IM}\cdot \text {V} - k^{\textrm{Delay}}_{\textrm{CXCL10}} \cdot C_{\textrm{CXCL10}}^{(1)} \nonumber \\ C_{\mathrm {CXCL10\_out}}^{(1)}&= k^{\textrm{Delay}}_{\textrm{CXCL10}} \cdot C_{\textrm{CXCL10}}^{(1)} \nonumber \\ \frac{d \text {CXCL10}}{dt}&= k_{\text {CXCL10}\_\text {IM}}\cdot \text {IM} +C_{\mathrm {CXCL10\_out}}^{(1)} -d_\text {CXCL10} \cdot \text {CXCL10}\nonumber \\ d_\text {CXCL10}&= \frac{ k_{\text {CXCL10}\_\text {IM}}\cdot \text {IM}_0}{\text {CXCL10}_0} \end{aligned}$$

As for CCL8, we used single-cell gene-expression of CXCL10 in macrophages as comparative data for the modelled CXCL10 production. This is achieved by the following equation.

20 $$\begin{aligned} \text {CXCL10}_\text {prod} =&\frac{k_{\text {CXCL10}\_\text {IM}}\cdot \text {IM} +C_{\mathrm {CXCL10\_out}}^{(1)}}{k_{\text {CXCL10}\_\text {IM}}\cdot \text {IM}_0} \end{aligned}$$

### Numerical methods for simulation

Differential equations are implemented in MATLAB 9.6.0.1072779 (R2019a) using the SIMULINK toolbox (The MathWorks Inc., Natick, MA, USA). Numerical solutions of the differential equation system are obtained using the variable step solver from Adams and Bashford (ode113, SIMULINK toolbox).

### Data

Model simulations are compared with data from Syrian hamsters comprising time series of monocytic macrophages, activated T/NK cells, B cells, type 2 alveolar epithelial cells, single-cell derived CXCL10 gene-expression by macrophages, CCL8 gene-expression by epithelial cells, neutralizing serum antibodies, pulmonary IgM levels, virus titers in lung homogenates, and viral RNA content in epithelial cells.

### Experiments

#### Ethics statement

The here described animal studies, including all animal protocols were approved by the regulatory state authority named “Landesamt für Gesundheit und Soziales” from Berlin in Germany (permit number 0086/20). All animal experiments were performed in accordance with appropriate guidelines. In agreement with the 3R principle, no additional animal experiments were conducted solely for this study. All experimental data were derived from our previous publications and are reported in accordance with the ARRIVE guidelines^29,39^.

#### Animal husbandry

Female and male Syrian hamsters (Mesocricetus auratus; RjHan:AURA, Janvier Labs, Saint-Berthevin, France) were housed in a BSL-3 facility in individually ventilated cages (IVCs; Tecniplast, Buguggiate, Italy) with bountiful enrichment (Carfil, Oud-Tunrhout, Belgium) and ad libidum access to food and water. Cage temperature and relative humidity were recorded daily and ranged from 22 to 24 °C and 40–55%, respectively. A minimum of 7 days was allowed for acclimatization of animals prior to start of experiments.

#### Virus stocks and PFU determination

SARS-CoV-2 isolate (BetaCoV/Germany/BavPat1/2020)^64^, was kindly provided by Drs. Daniela Niemeyer und Christian Drosten, Charité Berlin, Germany. Virus stocks for animal experiments were obtained by propagating virus under BSL-3 conditions on Vero E6 cells (ATCC CRL-1586) in minimal essential medium (MEM; PAN Biotech, Aidenbach, Germany) supplemented with 10% fetal bovine serum (FBS, PAN Biotech), 100 IU/mL penicillin G and 100 μg/mL streptomycin (Carl Roth, Karlsruhe, Germany). Prior to animal experiments, low passage stocks were titrated on Vero E6 cells under semi-solid overlay medium as described^65^. Briefly, Vero E6 cells were incubated with serial 10-fold virus stock dilutions for 2 hours. Following this, the virus inoculum was replaced by an overlay medium comprised of Dulbecco’s modified Eagle’s medium (DMEM, PAN Biotech, Aidenbach, Germany), 2.5% microcrystalline cellulose (Avicel RC-591, DuPont, Wilmington, DE, USA) and 10% FBS. After seventy-two hours of incubation at 37 °C in 5% CO2 atmosphere, cells were fixed with 4% formaldehyde for 24 hours and plaques were visualized by crystal violet counterstaining. Sequence integrity of virus stocks was determined by Illumina sequencing as described^66^ and aligned against the isolate reference sequence (GenBank: MT270101 and GISAID: EPI_ISL_406862). To determine viral burden, lung homogenates were stained with crystal violet and plaques are counted by eye as described in^39^.

#### Animal infection and collection of materials

10- to 12-week old female and male Syrian hamsters (Mesocricetus auratus; breed RjHan:AURA, Janvier Labs, France) were intranasally infected with $1\times 10^5$ plaque forming units (pfu) SARS-CoV-2 (variant B1, isolate BetaCoV/ Germany/ BavPat1/ 2020) under anesthesia as described previously^67^. Twice-daily clinical scoring of hamsters was performed to prevent any prolong suffering. Animals with >15% body weight loss for over 48 h were euthanized in accordance with the animal use protocol. A total of 39 hamsters were evaluated for our modelling distributed over the following experimental groups: (1) Control subjects (n=3), (2) SARS-CoV-2 infected subjects at four time points (2-, 3-, 5- and 14 p.i.), six animals per time point (n=24), (3) Mock-infected subjects at these time points, three animals per time point (n=12).

Timepoints of measurements were chosen to capture the early immune response as best as possible but also limiting the number of animals. We focussed on the earlier inflammatory phase characterized by higher dynamics of viral load, cell recruitment and activation. Viral load already dropped at d5, while re-convalescence was achieved at d14. Details of the choice of measurement points are explained in^39^. Euthanasia prior analysis occurred by cervical dislocation and exsanguination under anesthesia as previously described^29^. Among other materials, 1 ml cardiac blood (anticoagulated with EDTA) and lung lobes were collected for down-stream analyses. Specifically, the left lobe was used for histopathology, the right caudal lobe for single-cell analysis, the right cranial lobe for virological measurements and the right medial lobe for bulk RNA and proteomics analysis as described^39^. 3 naive and 12 mock-infected animals were combined for IgM measurements at t=0. Additional, proteomics measurements of 22 animals (d2:6, d3:5, d5:6, d14:5) were performed. For viral load determination, material of 6 infected hamsters per time point (2,3,5,14) was included. For scRNAseq and serum neutralization experiments, only 3 hamsters per time point (0,2,3,5,14) were analysed.

#### Single cell isolation from hamster lungs

Established cell isolation protocols were modified to suit BSL3 facility regulations. *Lobus caudalis* of the right lung was stored in 1$\times$ PBS, 0.5 % BSA containing 2 μg/ml ActinomycinD for single cell isolation. The lobes were dissociated mechanically and enzymatically, 2 min of clapping with tweezer in specific digestion medium (3,4 mg/ml Collagenase Cls II (Merck), 1 mg/mL DNase I (PanReac AppliChem), in 2 mL Dispase medium per lung lobe (Corning), 50 Caseinolytic Units/mL was executed, followed by 30 min incubation at 37 °C and 5% CO_2_. Next the cell suspension were further dissociated by pipetting and filtered through 70 μm cell strainers. Suspensions were spun at 350 $\times$ *g* for 6 min at 4 °C, pellets were subjected to red blood cell lysis by resuspension in corresponding buffer (BioLegend). The reaction was interrupted by washing with PBS/BSA buffer and cells were spun down by centrifugation. Prior subjection to 10$\times$ chips, cells were resuspended in low-BSA buffer ($1\times$ PBS, 0.04 % BSA), followed by filtration with 40 μm FloMi filters (Merck), cell number and viability determination was performed microscopically in trypan blue.

#### Single cell RNA expression quantification and assignment of cell types and proteomics

RNA isolation, sequencing and processing of and quantification of gene expression was done as described previously^39^. Filtered cells were adjusted to a final concentration of ca. 1000 cells/μL in 1$\times$ PBS with 0.04% BSA. 3’ GEM, Library & Gel Bead Kit v3.1 was used to partition cells into Gel-Beads-in-Emulsions (GEMs), aiming to recover 6000 single cells per hamster and organ. These single-cell libraries (quantified with Qubit, ThermoFisher, quality check with Agilent) were then sequenced on a Novaseq 6000 device (Illumina), with SP or S1 flow cells (read1: 28 nucleotides, read2: 64 nucleotides). Raw single-cell sequencing data were processed using CellRanger 3.1.0, and raw feature barcode matrices were read into Seurat. Cells were subjected to quality control, normalized, and integrated via function IntegrateData to eliminate batch effects, then subjected to PCA and UMAP dimensional reduction analyses, as well as Louvain clustering. Cell types were assigned through a combination of marker expression and label transfer from available mouse and human datasets. The expression of cell type-specific activation markers was quantified using Seurat’s DotPlot function with scaling of the data.

Marker genes of CD4+ T cells (Cd3e+Cd4+), CD8+ T cells (Cd3e+Cd8a+), NK cells (Cd3e-Nkg7+), monocytic macrophages (Ccr2+, Ccr5+, Arg1+), B cells (Cd79b+, Ms4a1+), and alveolar epithelial cells type 2 (Lamp3+) defined the Seurat cluster and thus the proportion of individual cell types. Cell counts of monocytic macrophages, activated (Gzma+ or Ifng+) T—and NK cells, B cells and type 2 alveolar epithelial cells are calculated as proportion of total cell number per lung lobe. Thereby, GZMA positive CD8+ and IFNG positive CD4+ cells are considered as proxies to assess immune response. Production of chemokines CCL8 and CXCL10 was determined as percent expressed in cluster.

IgM heavy chain was determined by using LC-MS/MS proteomics. Lung tissue was added to lysis and inactivation buffer (RIPA) and boiled at 95 °C for 10 min. The samples were then stored at − 80 °C. The samples were thawed on ice, the volume was adjusted to 50 μl with water and 25 μl of 50 U benzonase, 50 mM ABC, and 2 mM MgCl_2_ were added. This was followed by a 30-min incubation at 37 °C. Lysates were handled on a Biomek i7 workstation using the SP3 protocol with one-step reduction and alkylation. The samples were used for LC-MS/MS analysis.

#### Neutralizing antibodies

Serum neutralization was tested with serial dilutions (1:4 to 1:512) of inactivated serum, plated on sub-confluent monolayers of Vero E6 cells. 50 pfu SARS-CoV-2 were added per well and incubated for 72 h at 37 °C, fixed with 10% formalin for 24 h and stained with crystal violet. The highest effective dilution without cytopathic effect was counted.

### Estimation of parameters

We estimated 39 free model parameters by optimizing the agreement of simulation results and experimental data with the help of the following objective function.

21 $$\begin{aligned} \sum _{i=1}^m\bigg \{ \sum _{j=1}^{l}\bigg (\frac{f_{\textit{data}_i}(t_j)-\ln (f_{\textit{model}_i}(t_j,{\textbf {k}})\cdot {{C_i}_{\text {nor}}})}{\sigma _i}\bigg )^2\bigg \} \rightarrow \underset{{\textbf {k}}}{\min } \end{aligned}$$

Here, $f_{\textit{model}_i}(t_j,{\textbf {k}})$) denotes simulation results of variable *i*; $f_{\textit{data}_i}(t_j)$ are the means of logarithmized measurements of quantity *i* at time points $t_j$. Since we see no connection between signal strength and standard deviation, we average the standard deviation of the individual variables over the measurement points, where $\sigma _i$ is the mean of standard deviations of the logarithmized measurements of variable *i* at time points $t_j$. ${\textbf {k}}=k_1, \ldots k_n$ are the model parameters. Fitness function results of the different variables are summarized. For calculation of the fitness function of EU and EI, the same data of frequencies (%) of SARS-CoV-2 positive cells amongst alveolar epithelial cells type 2 were used. Since we want to avoid the same data contribute twice in the calculation of the summarized objective function, we use weighting factors of 0.7 for EI respectively 0.3 for EU. ${C_i}_{\text {nor}}$ are geometric means of measured quantities that resulted from data of pooled control groups. As in our previous work^49^, the optimization problem is approximately solved using (1+3)-evolutionary-strategies with self-adapting mutation step size^68,69^. Opimization was stopped after 200 generations without improvements. To increase the chance of finding the global optimum, we started optimization multiple times with different initial parameter settings.

Confidence intervals of parameter estimates are determined with the bootstrap method described in^70–72^. In brief, for each data point, we created a virtual random data point using the distribution of measurements and fitted parameters to these virtual time series data. Based on 1000 repetitions, we determined confidence ranges of the resulting parameter estimates. We consider parameters as well identifiable if the 95%-confidence interval is confined within a range less than one order of magnitude.

We estimated the sensitivity of each parameter by modifying their individual values by $\pm 10\%$ keeping all other parameters constant. Then, deteriorations of the objective function were studied. Results are shown in S1_File, Figure S1. Sensitivity is interpreted in a relative way, i.e., by comparing them between parameter estimates. Figure S2 in S1_File shows changes in the objective function, if two parameters are deflected simultaneously by $\pm 10\%$.

We determined bivariate correlations between parameter estimates by drawing from their univariate 95% confidence intervals (N=200 repetitions) and discarding values resulting in a significant deterioration of the likelihood (likelihood ratio test). Remaining parameter combinations were used to calculate the correlation of the respective parameter estimates.

Finally, to assess overall model fitting, we simulated scenarios with and without infective event and calculated values of the fitness function, Bayesian information criterion and Akaike information criterion using the same data of infected animals (Table S3 in S1_File).

## Supplementary Information

Supplementary Information 1.

Supplementary Information 2.

## Data Availability

All relevant data are within the manuscript and its Supporting Information file S2_File.xlsx. Sequencing data are available at https://www.ncbi.nlm.nih.gov/geo/query/acc.cgi?acc=GSE162208.

## Code availability

The R code for initial sequencing analysis is available at github.com, https://github.com/Berlin-Hamster-Single-Cell-Consortium/Single-cell-sequencing-of-COVID-19-pathogenesis-in-golden-Hamsters. Code of our model will be made publicly available after acceptance.
