# Supplementary material for: A biomathematical model of SARS-CoV-2 in Syrian hamsters
Source: Sci Rep. 2024 Dec 18;14:30541. doi: 10.1038/s41598-024-80498-9 (PMC11655638; doi:10.1038/s41598-024-80498-9)
Supplement: Supplementary file 1 — Supplementary Information 1. [file 41598_2024_80498_MOESM1_ESM.pdf]

A biomathematical model of SARS-CoV-2 in Syrian hamsters - supplement material

Sibylle Schirm[1*], Geraldine Nouailles[2], Holger Kirsten[1], Jakob Trimpert[3], Emanuel Wyler[4], Luiz Gustavo Teixeira Alves [4], Markus Landthaler [4,5], Peter Ahnert[1], Norbert Suttorp[2], Martin Witzenrath[2,6], Markus Scholz[1]

1 Institute for Medical Informatics, Statistics and Epidemiology, University of Leipzig, Leipzig, Germany
2 Charité – Universitätsmedizin Berlin, Corporate Member of Freie Universität Berlin and Humboldt-Universität zu Berlin, Department of Infectious Diseases and Respiratory Medicine and Critical Care, Berlin, Germany
3 Institute of Virology, Freie Universität Berlin, Berlin, Germany
4 Berlin Institute for Medical Systems Biology (BIMSB), Max Delbrück Center for Molecular Medicine in the Helmholtz Association (MDC), Berlin, Germany
5 Institute for Biology, Humboldt-Universität zu Berlin, Berlin, Germany
6 German Center for Lung Research (DZL), Berlin, Germany

*Corresponding author: sibylle.schirm@imise.uni-leipzig.de

Parameters and initial values

Table S1. Parameter estimates.

parameter	meaning	value	95% confidence intervals	
k_{EU}	regeneration rate of unaffected cells	1.291E+01	[7.822E+00 , 1.844E+01]	fitted
$k_{\mathrm{EU\_V}}$	infection rate of susceptible cells	3.510E-03	[3.265E-03 , 3.801E-03]	fitted
d_{EI}	removal rate of infected cells	4.508E+01	[4.199E+01 , 4.897E+01]	fitted
$k_{\mathrm{EA\_CD8^+}}$	removal rate of affected cells by $CD8^+$ T cells	4.874E-04	[2.435E-04 , 9.871E-04]	fitted
$k_{\mathrm{EA\_NK}}$	removal rate of affected cells by NK cells	4.081E-02	[1.860E-02 , 9.027E-02]	fitted
d_{IM}	influx rate of activated macrophages	2.177E-01	[1.400E-01 , 2.978E-01]	fitted
$k_{\mathrm{IM\_V}}$	increased influx of IM due to virus presence	1.156E-03	[9.047E-04 , 1.949E-03]	fitted
k_{V}	virus replication rate	1.905E+03	[1.764E+03 , 2.050E+03]	fitted
$k_{\mathrm{V\_AB}}$	depletion of virus by neutralizing antibodies	1.589E-01	[1.463E-01 , 1.721E-01]	fitted
$k_{\mathrm{V\_IgM}}$	depletion of virus by IgM type antibodies	1.025E-02	[6.876E-03 , 1.504E-02]	fitted
d_{V}	virus decay	9.876E+00	[9.504E+00 , 1.280E+01]	fitted
$k_{\mathrm{V}}^{\mathrm{Delay}}$	delay of cell reaction due to virus presence	6.332E-01	[4.234E-01 , 9.198E-01]	fitted
$k_{\mathrm{CD8^+\_CXCL10}}$	attraction of $CD8^+$ T cells by CXCL10	3.283E-02	[2.256E-02 , 4.911E-02]	fitted
$k_{\mathrm{CD8^+\_CCL8}}$	attraction of $CD8^+$ T cells by CCL8	4.614E-01	[3.047E-01 , 6.431E-01]	fitted
$k_{\mathrm{CD8^+\_V}}$	activation of $CD8^+$ T cells by virus	3.910E-02	[2.525E-02 , 6.078E-02]	fitted
$k_{\mathrm{CD4^+\_CXCL10}}$	attraction of $CD4^+$ T cells by CXCL10	1.073E-02	[7.330E-03 , 1.587E-02]	fitted
$k_{\mathrm{CD4^+\_CCL8}}$	attraction of $CD4^+$ T cells by CCL8	5.148E-01	[3.547E-01 , 7.573E-01]	fitted
$k_{\mathrm{CD4^+\_V}}$	activation of $CD4^+$ T cells by virus	5.695E-03	[3.739E-03 , 8.415E-03]	fitted
$k_{\mathrm{B\_CXCL10}}$	attraction of B cells by CXCL10	5.113E-02	[3.368E-02 , 7.609E-02]	fitted
$k_{\mathrm{CXCL10\_IM}}$	production of CXCL10 by IM	2.507E+00	[9.636E-01 , 4.394E+00]	fitted
$k_{\mathrm{CXCL10\_IM\_V}}$	additional CXCL10 production by IM due to virus presence	3.479E-02	[1.875E-02 , 8.178E-02]	fitted
$k_{\mathrm{CXCL10}}^{\mathrm{Delay}}$	delay of additional CXCL10 production by IM	3.537E-01	[2.179E-01 , 6.004E-01]	fitted

Continued on next page

Table S1 – continued from previous page

parameter	meaning	value	95% confidence intervals	
	due to virus presence			
k_{CCL8_EA}	CCL8 production by EA	2.147E+01	[1.118E+01 , 3.763E+01]	fitted
$k_{CCL8\_EI\_V}$	CCL8 production by EI	1.359E+02	[7.882E+01 , 2.852E+02]	fitted
k_{CCL8}^{Delay}	delay of CCL8 production by EI	2.716E-01	[1.895E-01 , 3.987E-01]	fitted
k_{IgM}	IgM antibody production	1.388E+00	[9.369E-01 , 2.133E+00]	fitted
k_{IgM_V}	additional IgM antibody production	7.496E-01	[5.040E-01 , 1.111E+00]	fitted
	due to virus presence	0.000E+00		
Z_{IgM}^{max}	maximum of IgM production	2.269E+00	[1.083E+00 , 4.992E+00]	fitted
Z_{IgM}^{nor}	IgM production in steady-state	1.161E-01	[4.832E-02 , 2.233E-01]	fitted
Z_{IgM}^{b}	sensitivity of IgM production regarding	9.043E-02	[3.907E-02 , 1.942E-01]	fitted
	virus presence			
Z_{IgM}^{min}	minimum of IgM production	0.000E+00		set
k_{B_PC}	maturation rate of antibody affinity	1.740E-03	[1.002E-03 , 2.152E-03]	fitted
d_{PC}	decay of plasma cells	6.001E-01	[4.034E-01 , 8.696E-01]	fitted
k_{AB}^{Delay}	delay of plasma cell maturation	8.003E+01	[3.649E+01 , 1.725E+02]	fitted
k_{NK_CCL8}	NK recruitment by CCL8	7.073E-01	[4.610E-01 , 1.151E+00]	fitted
k_{NK_CXCL10}	NK recruitment by CXCL10	2.883E-03	[1.934E-03 , 4.204E-03]	fitted
k_{NK_V}	NK recruitment due to virus presence	3.031E-05	[1.976E-05 , 4.635E-05]	fitted
Z_{AB}^{max}	maximum AB production by plasma cells	6.443E+01	[4.378E+01 , 9.272E+01]	fitted
Z_{AB}^{nor}	AB production by plasma cells in steady-state	3.869E-01	[2.608E-01 , 5.430E-01]	fitted
Z_{AB}^{b}	steepness of increase in AB production	5.818E+00	[3.515E+00 , 7.666E+00]	fitted
	by plasma cells			
Z_{AB}^{min}	minimum AB production by plasma cells	0.000E+00		set
d_{AB}	waning of neutralizing antibodies	1.000E-02	[2.137E-03 , 3.935E-02]	set
d_{NK}	decrease of NK	7.102E-01		set
d_{CD4^+}	decrease of $CD4^+$ T cells	5.255E-01		set
d_{CD8^+}	decrease of $CD8^+$ T cells	4.942E-01		set
d_{CXCL10}	decrease of CXCL10	2.507E+00		set
d_{B}	decrease of B cells	5.113E-02		set
d_{CCL8}	decrease of CCL8	2.147E+01		set
d_{IgM}	decrease of IgM	1.195E-01		set
d_{EA}	decrease of EA	1.278E+03		set
P_{EU}	production of EU	1.282E+03		set

Table S2. Initial values and normal values. Initial parameters of model state parameters are typically set. Steady-state values are derived from the data and are multiplied with the modeled state parameter to allow comparisons with the data.

EU_0	initial percentage of unaffected epithelial cells [%]	9.900E+01
EI_0	initial percentage of infected epithelial cells [%]	0.000E+00
EA_0	initial percentage of activated epithelial cells [%]	1.000E+00
V_0	initial virus load (virus titers in lung homogenates per g lung)	0.000E+00
AB_0	initial concentration of neutralizing antibodies	0.000E+00
$CD8^+_{A0}$	initial value of $CD8^+$ T cells (set)	1.000E+00
$CD8^+_{A\mathrm{nor}}$	$CD8^+$ steady-state value of T cells [cells per lung lobe] from data	1.812E+04
$CD4^+_{A0}$	initial value $CD4^+$ T cells (set)	1.000E+00
$CD4^+_{A\mathrm{nor}}$	$CD4^+$ steady-state value of T cells value [cells per lung lobe] from data	4.095E+04
B_0	initial value of B cells (set)	1.000E+00
B_{nor}	steady-state value of B cells [cells per lung lobe] from data	1.083E+06
NK_{A0}	initial value of NK cells (set)	1.000E+00
$NK_{A\mathrm{nor}}$	steady-state value of NK cells [cells per lung lobe] from data	2.252E+05
IgM_0	initial value of IgM type antibodies (set)	1.000E+00
IgM_{nor}	steady-state value IgM type antibodies from data	9.134E+00
IM_0	initial value of inflammatory macrophages (set)	1.000E+00
IM_{nor}	steady-state value of inflammatory macrophages [cells per lung lobe] from data	1.816E+06
$CXCL10_0$	initial value of CXC chemokine ligand 10 (set)	1.000E+00
$CCL8_0$	initial value of chemokine CCL8 (set)	1.000E+00
PC_0	initial value of plasma cells (set)	0.000E+00

Sensitivity analysis

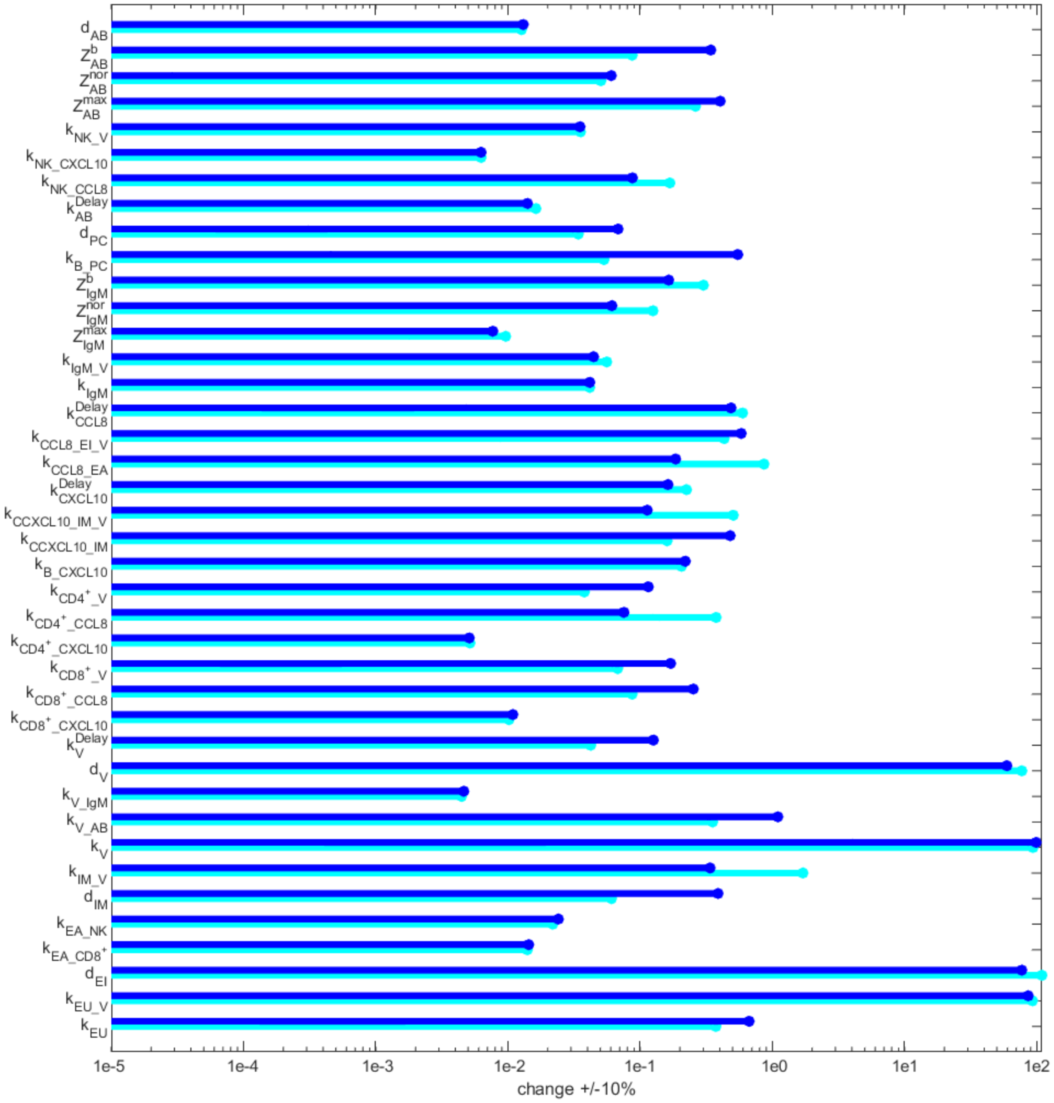

Fig S1. Parameter sensitivity when changing a single parameter. The horizontal bars show the deterioration of the objective function (in %) when the parameter value is multiplied by the factor 0.9 (cyan) or 1.1 (blue).

Table S3. Quantification of model fitness. We present values of the fitness function (residual sum of squares) for the different compartments with available data. We compare the fitness of the model with infection at day zero with that of a null model without infections. BIC and AIC denote Bayesian information criterion respectively Akaike information criterion.The Bayes factor between our model and the constant model is decisive

variable	infection model	constant model
V	9.41E-01	5.85E+01
EI	2.93E+00	4.85E+01
IgM	9.42E-01	1.07E+01
AB	3.47E+00	1.65E+02
NK cells	3.88E+01	2.24E+02
IM cells	1.60E+01	7.09E+01
CD8 cells	5.67E+00	1.25E+02
CD4 cells	6.44E+00	1.12E+02
B cells	2.31E+00	6.85E+00
CXCL10IMprod	3.81E+01	3.15E+02
CCL8prod	1.81E+01	1.31E+02
sum of fitness functions	1.34E+02	1.25E+03
BIC	1.29E+02	3.04E+02
AIC	5.45E+01	2.29E+02

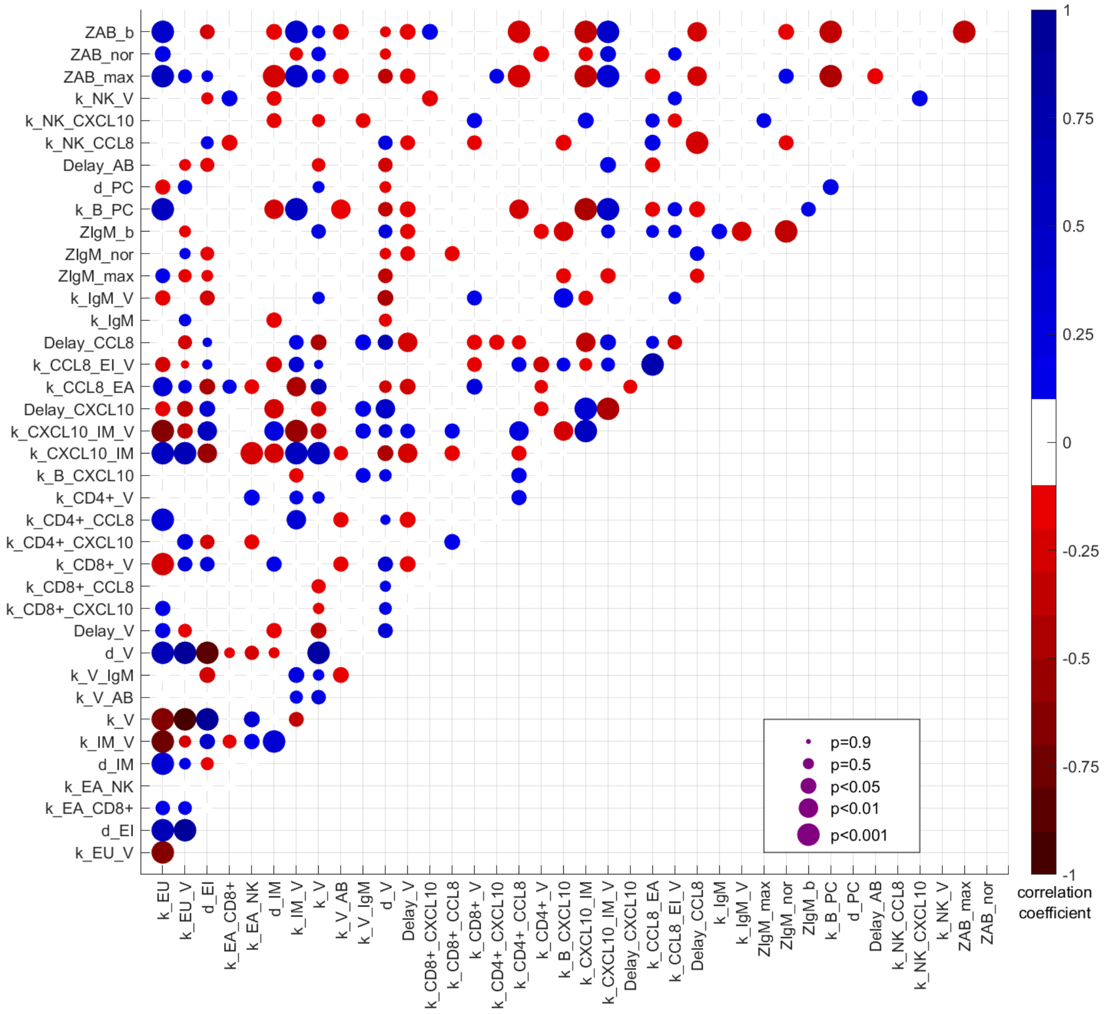

Fig S2. Correlation of parameter estimates. We present the results of our correlation analysis of parameter estimates. Estimated Pearson correlations between two parameter estimates are color-coded (red=negative correlations, blue=positive correlations).
